# Protease and gag diversity and drug resistance mutations among treatment-naive Mexican people living with HIV

**DOI:** 10.1186/s12879-022-07446-8

**Published:** 2022-05-10

**Authors:** Samantha Climaco-Arvizu, Víctor Flores-López, Carolina González-Torres, Francisco Javier Gaytán-Cervantes, María Concepción Hernández-García, Paola Berenice Zárate-Segura, Monserrat Chávez-Torres, Emiliano Tesoro-Cruz, Sandra María Pinto-Cardoso, Vilma Carolina Bekker-Méndez

**Affiliations:** 1 Unidad de Investigación Médica en Inmunología e Infectología, Hospital de Infectología “Dr Daniel Méndez Hernández”, Centro Médico Nacional “La Raza”, Instituto Mexicano del Seguro Social (IMSS), Ciudad de México, C.P. 02990 México; 2 Laboratorio de Medicina Traslacional, Instituto Politécnico Nacional, Ciudad de México, México; 3 Department of Biochemistry, University of Cambridge, Cambridge, UK; 4 División de Desarrollo de La Investigación, Instituto Mexicano del Seguro Social, Ciudad de México, México; 5 Instituto Mexicano del Seguro Social (IMSS), Hospital de Infectología “Dr Daniel Méndez Hernández”, Centro Médico Nacional (CMN), La Raza”, Ciudad de México, México; 6 Centro de Investigación en Enfermedades Infecciosas, Instituto Nacional de Enfermedades Respiratorias Ismael Cosío Villegas, Ciudad de México, C.P. 14080 México

**Keywords:** Human immunodeficiency virus, Antiretroviral therapy, HIV drug resistance mutations, HIV genotyping, Gag, Protease, Next generation sequencing

## Abstract

**Introduction:**

In Mexico, HIV genotyping is performed in people living with HIV (PLWH) failing their first-line antiretroviral (ARV) regimen; it is not routinely done for all treatment-naive PLWH before ARV initiation. The first nationally representative survey published in 2016 reported that the prevalence of pretreatment drug mutations in treatment-naive Mexican PLWH was 15.5% to any antiretroviral drug and 10.6% to non-nucleoside reverse transcriptase inhibitors (NNRTIs) using conventional Sanger sequencing. Most reports in Mexico focus on HIV *pol* gene and nucleoside and non-nucleoside reverse transcriptase inhibitor (NRTI and NNRTI) drug resistance mutations (DRMs) prevalence, using Sanger sequencing, next-generation sequencing (NGS) or both. To our knowledge, NGS has not be used to detect pretreatment drug resistance mutations (DRMs) in the HIV protease (PR) gene and its substrate the Gag polyprotein.

**Methods:**

Treatment-naive adult Mexican PLWH were recruited between 2016 and 2019. HIV Gag and protease sequences were obtained by NGS and DRMs were identified using the WHO surveillance drug resistance mutation (SDRM) list.

**Results:**

One hundred PLWH attending a public national reference hospital were included. The median age was 28 years-old, and most were male. The median HIV viral load was 4.99 [4.39–5.40] log copies/mL and median CD4 cell count was 150 [68.0–355.78] cells/mm^3^. As expected, most sequences clustered with HIV-1 subtype B (97.9%). Major PI resistance mutations were detected: 8 (8.3%) of 96 patients at a detection threshold of 1% and 3 (3.1%) at a detection threshold of 20%. A total of 1184 mutations in Gag were detected, of which 51 have been associated with resistance to PI, most of them were detected at a threshold of 20%. Follow-up clinical data was available for 79 PLWH at 6 months post-ART initiation, seven PLWH failed their first ART regimen; however no major PI mutations were identified in these individuals at baseline.

**Conclusions:**

The frequency of DRM in the HIV protease was 7.3% at a detection threshold of 1% and 3.1% at a detection threshold of 20%. NGS-based HIV drug resistance genotyping provide improved detection of DRMs. Viral load was used to monitor ARV response and treatment failure was 8.9%.

**Supplementary Information:**

The online version contains supplementary material available at 10.1186/s12879-022-07446-8.

## Introduction

In 2021, the National Centre for AIDS Prevention and Control (CENSIDA) reported that 195,860 people were living with Human Immunodeficiency Virus (PLWH) in Mexico. Of these, 113,788 (58.1%) were prescribed antiretroviral therapy (ART) and of those, around 20% did not achieve sustained viral suppression [1, 2]. According to the Mexican National Guidelines, the recommended first-line ART regimen for treatment-naive adults consists of two nucleoside reverse-transcriptase inhibitors (NRTIs) and an integrase inhibitor (INSTI) [3]. As an alternative, the use of a non-nucleoside reverse-transcriptase inhibitor (NNRTI) or boosted protease inhibitor (PI) is recommended [3]. Despite the effectiveness of ART and considerable efforts to help control the HIV/AIDS epidemic by 2030 (95–95-95), ART failure due to drug resistance mutations (DRMs) is proving a challenge for ART provision and HIV care [4]. In 2017, the World Health Organization (WHO) published a report on HIV drug resistance addressing the alarming increase in the prevalence of DRMs in individuals initiating their first-line ART regimen, linking DRMs to treatment failure [5]. According to the 2018 Recommendations of the International Antiviral Society (IAS)–USA Panel, HIV resistance testing is recommended for all individuals with HIV infection who are newly diagnosed, before they initiate ART and in PLWH with ART failure [6]. Genotyping DNA-based assays are the most widely used for HIV DRMs detection. Next-generation sequencing (NGS) improves the detection of DRMs, by yielding substantially more throughput with higher sensitivity and reproducibility compared to conventional Sanger sequencing [7, 8]. In Mexico, HIV genotyping is performed in PLWH failing their first-line regimens; it is not routinely performed for all PLWH who are treatment-naive and starting their first-line ART [9]. Several studies have reported the prevalence of HIV DRMs in treatment-naive Mexican PLWH [10–12]. According to a nationally representative survey, in Mexico the prevalence of any antiretroviral (ARV) resistance drug in treatment-naive PLWH is greater than 10%. Also, this report concluded that PWLH who initiated with NNRTI-based regimens achieved significantly lower levels of viral suppression compared to those who initiated with PI-based regimens [11]. To our knowledge, NGS has not be used to detect DMRs in the protease (PR) gene and its substrate the Gag polyprotein in ART-naive Mexican PLWH. Drug resistance to PI arises by the accumulation of major mutations in the PR, mainly in the active site pocket (V32I, L33F, M46I, I47V, I54L/M, V82A/F and I84V) [13]. These DRMs confer resistance to most PI, among them the most commonly used: atazanavir/r (ATV/r), lopinavir/r (LPV/r) and darunavir/r (DRV/R) [14]. Besides the DRMs directly affecting the PR, DRMs to PI have also been studied in the natural substrate, the Gag polyprotein [15]. The PR recognizes and cleaves Gag polyproteins into their fully active forms required for the maturation of HIV particles at the final stage of the viral cycle [16]. Gag cleavage (CS) and non-cleavage site (NCS) mutations are directly associated with drug resistance to PI and NRTI resulting in virologic failure [17, 18]. Gag NCS mutations R76K, Y79F and T81A in the Matrix (MA), I389T and I401V in the Nucleocapsid (NC) have been associated with virologic failure to LPV/r and Nelfinavir (NFV) respectively [19, 20]. Also, Gag mutations can directly confer resistance to PI, in the absence of detectable DRMs in the PR [15]. Therefore, many studies have proposed that a complete study of PI resistance, should include the full length of Gag in addition to protease [21, 22]. To our knowledge, this is the first study that uses NGS to detect the presence of PR and Gag DMRs in treatment-naive Mexican PLWH.

## Methods

### Study design and population

A cross-sectional study was conducted at the Immunology and Infectology Research Unit of Hospital de Infectología, Centro Médico Nacional La Raza (CMNR) del Instituto Mexicano del Seguro Social (IMSS), Mexico City, Mexico. One Hundred HIV-positive ART-naive adult PLWH (over 18 years) were recruited between 2016 and 2019. This study was performed in accordance with guidelines of the Helsinki Declaration and was approved by the Bioethical Committee of the IMSS National Research (CNIC-R-2017-785-106). Written informed consent was obtained from all participants before blood sample donation and data were processed using unique identifiers to ensure confidentiality. Baseline and follow-up clinical data, including CD4 cell count, plasma viral load (pVL), HIV diagnosis date, ART initiation date, first-line ART regimen, changes in the initial treatment regimen, time between HIV diagnosis and ART initiation were obtained from the individuals’ medical records. Demographic characteristics, including gender, age and risk factors for HIV infection were obtained by means of a validated questionnaire at the time of blood-sample donation.

### Blood collection, and RNA extraction

Approximately 4 ml of peripheral blood were collected from all PLWH in ethylenediaminetetraacetic acid (EDTA) Vacutainer tubes (BD, San Jose, CA), and immediately processed to separate plasma by centrifugation at 2,500 rpm for 10 min at room temperature. Plasma samples were apportioned into 1.0 mL aliquots and stored at − 70 °C until use. For RNA extraction, a 1 mL plasma aliquot was thawed per subject and centrifuged at 14,000 rpm at 4 °C for 2 h to concentrate HIV particles. RNA was extracted using the QIAamp Viral RNA Mini Kit (Qiagen, Hilden, Germany) according to the manufacturer’s protocol.

### Complementary DNA synthesis (cDNA), and polymerase chain reaction (PCR)

The coding region of HIV-1 Gag and Protease (HXB2 nucleotides 790 to 2550) was amplified using primers and conditions as published elsewhere [23, 24]. Briefly, extracted RNA was subjected to a one-step RT-PCR followed by a nested amplification. Primer sequences, reagents and cycling conditions are provided in (Additional file 1). Amplicons were visualized by electrophoresis on a 1% agarose gel stained with ethidium bromide under ultraviolet light to confirm expected amplicon size (1998 bp).

### Library construction and sequencing of HIV-1 Gag and protease

Amplicons were purified using the MinElute PCR purification kit (Qiagen, Hilden, Germany) and quantitated using Qubit ds DNA Assay Kit (ThermoFisher, Waltham, MA, USA) according to the manufacturers' instructions. DNA libraries were prepared using the Nextera XT DNA Sample Preparation Kit (Illumina, San Diego, CA, USA), using 1 ng of input DNA, and indexed using Nextera XT Index Kit (Illumina, San Diego, CA, USA), according to manufacturers’ instructions. Libraries were checked for size and molarity using the High sensitivity D1000 ScreenTape (Agilent, Santa Clara, CA, USA) on a 4200 TapeStation (Agilent, Santa Clara, CA, USA). DNA libraries were pooled at 4 nM and further diluted to 9 pM. Libraries were sequenced on a MiSeq platform (Illumina, Santa Clara, CA, USA) using 2 × 250 cycles (MiSeq Reagent Kits v.2) with 5% PhiX as a control.

### Sequence data analysis

In order to identify HIV Gag and Protease DRMs we took a three-fold approach, first we assembled full length sequences using automated Target Restricted Assembly Method **(**a TRAM, version 2.1.0) locus assembler, an iterative assembler that performs reference-guided local de novo assemblies using a variety of available methods [25], with Trinity (v2.9.0) [26] as the de novo assembler and the HIV-1 HXB2 sequence (GenBank: K03455) as the template. We also determined codon-specific variants as well as their relative frequencies using an alignment and variant-calling pipeline for Illumina deep sequencing of HIV-1, based on the probabilistic aligner profile Hidden Markov Model for HIV (HIVMMER, version 0.1.2) [27] with the HXB2 as reference sequence. Finally, we aligned the full length sequences to the HXB2 reference using Burrows-Wheeler Aligner (BWA, version 0.7.17), a software package for mapping low-divergent sequences against a large reference genome [28]. Phylogenetic trees were generated from sequences obtained from 96 treatment-naive Mexican PLWH. We identified two viral sequences originating from individual 37, located in different branches. HIV reference strains representing all HIV subtypes were used (Additional file 1). The nucleotide sequences were aligned using the multiple sequence alignment program Clustal-omega (version 1.2.1) [29] and edited manually using Seaview (version 5.0.4), a multiplatform program designed to facilitate multiple alignment and phylogenetic tree building from molecular sequence data through the use of a graphical user interface [30]. A neighbor joining distance tree was constructed for each region separately: gag (1683 nucleotides) and protease (303 nucleotides). In order to provide statistical support to the constructed trees, they were subjected to 10,000 bootstrap replicas. Trees were custom-drawn using the ETE toolkit version 3 [31]. To ensure accuracy, a detection threshold of 1% was used (based on the intrinsic error rate of the system [8]). To calculate sequencing depth we used Sequence Alignment/Map (SAM tools, version 1.10) [32]. We achieved high level depth, with a median sequencing of 7616.84 reads (interquartile range (IQR), 7543.73 -7793.41) for Gag and Protease respectively (Additional file 1).

### Drug resistance mutations (DRMs)

HIV Drug Resistance Database (HIVDB; version 8.8) and the International AIDS Society 2019 drug resistance mutations in HIV-1 list were used [33, 34]. Gag sequences were analyzed for the presence of specific mutations previously reported to be associated with resistance to PI and treatment failure; these were: E12K, V35I, E40K, G62R, L75R, R76K, Y79F, T81A K112E, G123E, M200I, H219Q/P, Q369H, V370A/M, I389T, V390A/D, R409K, E468K, Q474L, P497L; as well as mutations in Gag CS: V128I/T/A, Y132F, A360V, V362I, L363M/F/C/N/Y, S368C/N, S373P/Q, A374P/S, T375N/S, I376V, G381S, E428G, Q430R, A431V, K436E/R I437T/V, L449F/P/V, S451T/G/R, P452S/K and P453A/L/T [20, 35]. Different detection thresholds (1%, 2%, 5%, 10% and, 20%) were used to identify the prevalence of low abundance (or minority) mutations as these could be missed using standard clinically relevant threshold (20%, Sanger sequencing).

## Results

### Study cohort

**Fig. 1:**
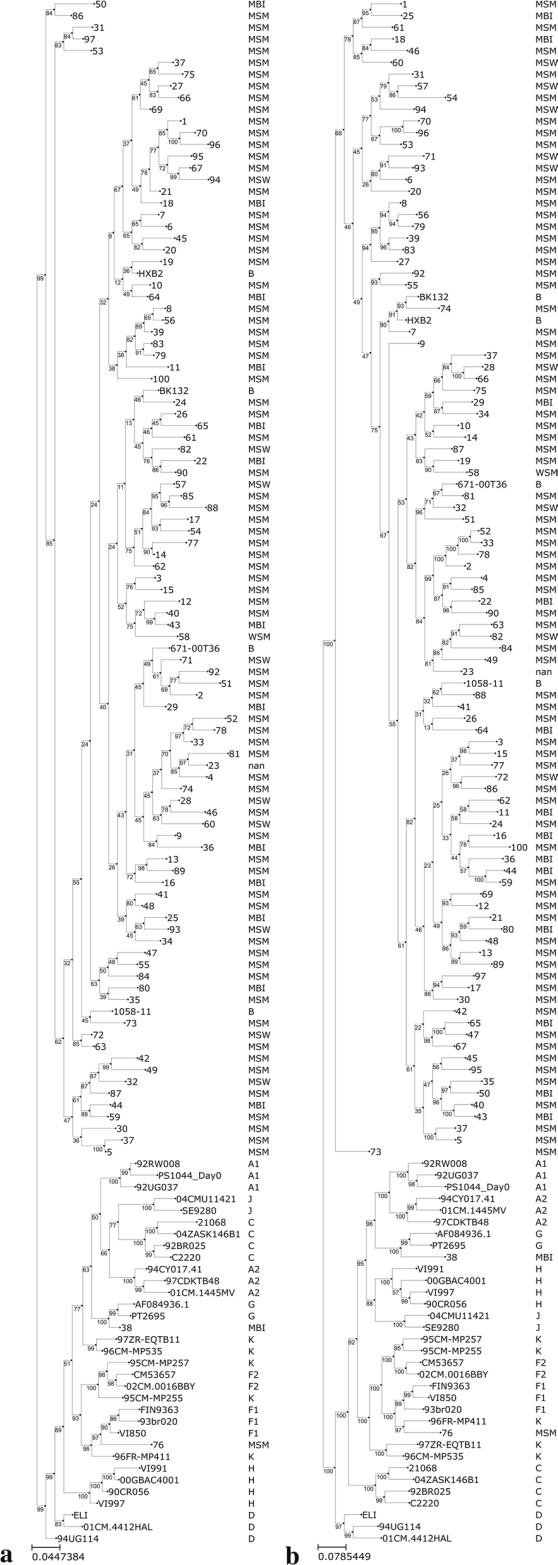
Phylogenetic tree analysis of HIV gag and protease from ART-naive Mexican individuals. Neighbor joining trees were constructed from the aligned sequences of **a** protease and **b** Gag. HIV risk factors are denoted by MSM (men who have sex with men), MSW (men who have sex with women), and MBI (men bisexual). The corresponding subgroup for each reference strain is shown next to the accession number

Demographic and clinical characteristics of our cohort are shown in Table 1. Hundred treatment-naive PWLH were enrolled. Their baseline median [interquartile range] pVL was 4.99 [IQR 4.39–5.40] log RNA copies/mL and their baseline median CD4 cell count was 150 [IQR 68.0–355.78] cells/mm^3^. HIV acquisition risk factor was predominantly men who have sex with men (74.0%) (Table 1). HIV sequences were assigned predominantly to HIV-1 subtype B (Fig. 1).

**Table 1:** Demographic and clinical characteristics of treatment-naive Mexican PLWH

**Variables**	
Demographics	
Gender, n (%)	
Male	99 (99.0%)
Female	1 (1.0%)
Age (years), median (min–max)	28 (18–66)
Marital status, n (%)	
Single	77 (77.0%)
Married	4 (4.0%)
Divorced	2 (2.0%)
Free union	16 (16.0%)
Widowed	1 (1.0%)
Literacy, n (%)^1^	
Primary school	3 (3.2%)
Junior High school	7 (7.4%)
High school	26 (27.4%)
Technical qualification	7 (7.4%)
Degree	50 (52.6%)
Postgraduate	2 (2.1%)
Employment, n (%)	
Employed	86 (86.0%)
Unemployed	1 (1.0%)
Student	13 (13.0%)
HIV	
CD4 count (cell/mm^3^), median [IQR]^2^	150[68.0–355.78]
CD4 cell count (%), mean (SD)^3^	11.7 (7.8)
Plasma viral load (copies/mL)^2^, median [IQR]	99,551 [24256.75–248,259.25]
Plasma viral load (log copies/mL)^2^, median [IQR]	4.99 [4.39–5.40]
HIV acquisition risk factor, n (%)^4^	
Men who have sex with men	74 (74.7%)
Heterosexual	11 (11.1%)
Bisexual	14 (14.1%)
HIV-1 subtype, n (%)	
B	94 (97.9%)
Non-B	2 (2.1%)
ART	
Time Dx to ART initiation (days), median [IQR]^2^	38.5 [20.8–60.5]
First-line regimen, n (%)^2^	
TDF-FTC-EFV	49 (50.0%)
TDF-FTC-ATVr	18 (18.4%)
ABC-3TC-ATVr	15 (15.3%)
ABC-3TC-EFV	12 (12.2%)
ABC-TDF-ATVr	1 (1.0%)
TDF-FTC-LPVr	1 (1.0%)
TDF-3TC-EFV	1 (1.0%)
ABC-3TC-DTG	1 (1.0%)

*ART* antiretroviral therapy, *TDF* Tenofovir, *FTC* Emtricitabine, *EFV* Efavirenz, *ATV/r* Atazanavir/ritonavir, *ABC* Abacavir, *3TC* Lamivudine, *LPV/r* Lopinavir/ritonavir, *DTG* Dolutegravir, *n* number, *%* percentage, IQR = interquartile range, *HIV* Human Immunodeficiency Virus, *Dx* diagnosis

^1^Data on literacy was missing for 5 individuals

^2^Data was missing for 2 individuals

^3^Data on CD4 cell count (%) was missing for 27 individuals

^4^Data was missing for 1 individual

### Residue variation of HIV Gag and protease

Full-length Gag and Protease sequences analysis revealed high variation in both genes (Table 2). Mutations in both Gag CS and NCS were identified. The distribution of all the identified mutations in Gag and protease are shown in (Additional file 1). We observed 1186 amino acid (aa) changes at 346 positions at full-length Gag, and 175 aa changes in 74 positions throughout the protease gene. The capsid protein (51.9%) contained the lowest number of polymorphic positions followed by nucleocapsid (78.2%), matrix (84.8%), and p6 (92.3%).

**Table 2:** HIV Gag and Protease identified mutations in PLWH naive to antiretroviral therapy (n = 96)

Protein		Length (aa)	Residues with at least one mutation n (%)	Total mutations observed
Gag		500	346 (69.2)	1186
	Matrix	132	112 (84.8)	433
	Capsid	231	120 (51.9)	221
	p2	14	11 (78.6)	72
	Nucleocapsid	55	43 (78.2)	153
	p1	16	12 (75.0)	35
	p6	52	48 (92.3)	272
Protease		99	74 (74.7)	175

*aa* amino acids, *n* number, *%* percentage, HIV: human immunodeficiency virus, PLWH: people living with HIV

### Drug resistance mutations (DRMs)

**Fig. 2:**
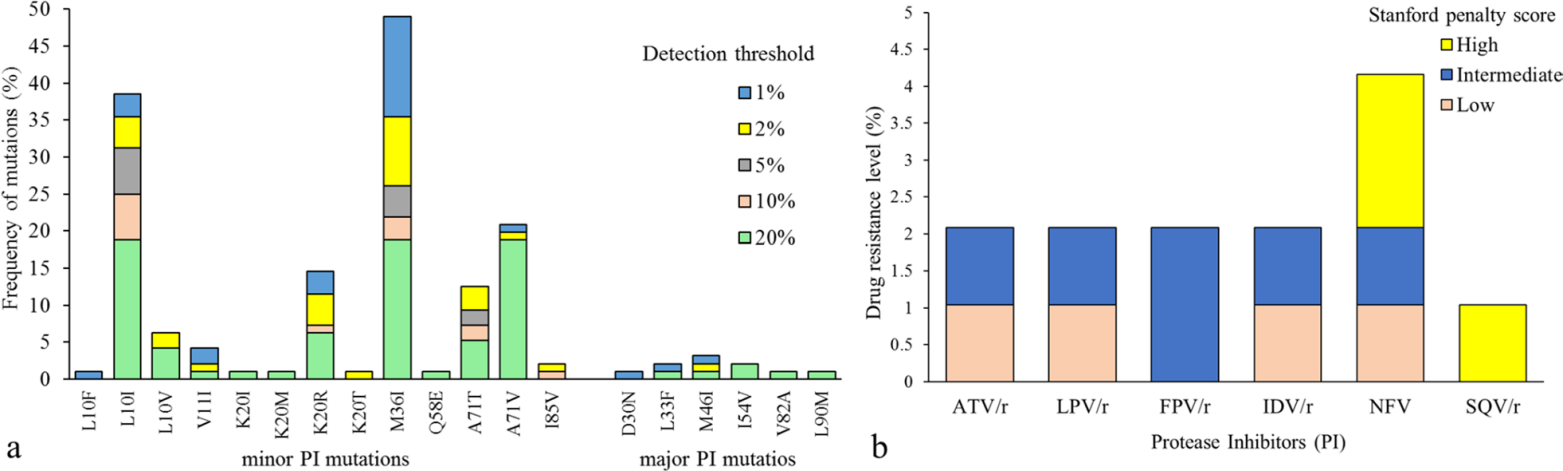
HIV Protease drug resistance mutation frequency and levels of PI resistance. **a** Frequency of minor and major PI resistance mutations identified with NGS at different detection sensitivity thresholds in patients without experience of antiretroviral therapy (n = 96). **b** Levels of resistance to protease inhibitors calculated with the Stanford HIVdb program using the 20% threshold. *ATV/r* ritonavir-boosted atazanavir. LPV/r = ritonavir-boosted lopinavir. *FPV/r* ritonavir-boosted fosamprenavir. IDV/r = ritonavir-boosted indinavir. *NFV* nelfinavir. *SQV/r* ritonavir-boosted saquinavir

Major and minor PI drug resistance mutations were detected (Fig. 2). Major PI resistance mutations were found in 8 (8.3%) of 96 patients at a detection threshold of 1% and 3 (3.1%) at a detection threshold of 20%. The most common major resistance mutation was M46I at 3.1% (3/96) frequency at 1% threshold and I54V with 2.1% (2/96) frequency at 20% threshold (Fig. 2a). Several minor PI resistance mutations were identified, M36I was the most frequent with 50.0% (48/96) and 19.8% (19/96) frequency at 1% and 20% detection threshold, respectively. Inferred levels of resistance to PI were determined, intermediate resistance was found in 2.08% (2/96) for both atazanavir and lopinavir (Fig. 2b).

### Gag mutations associated with resistance to PI

**Fig. 3:**
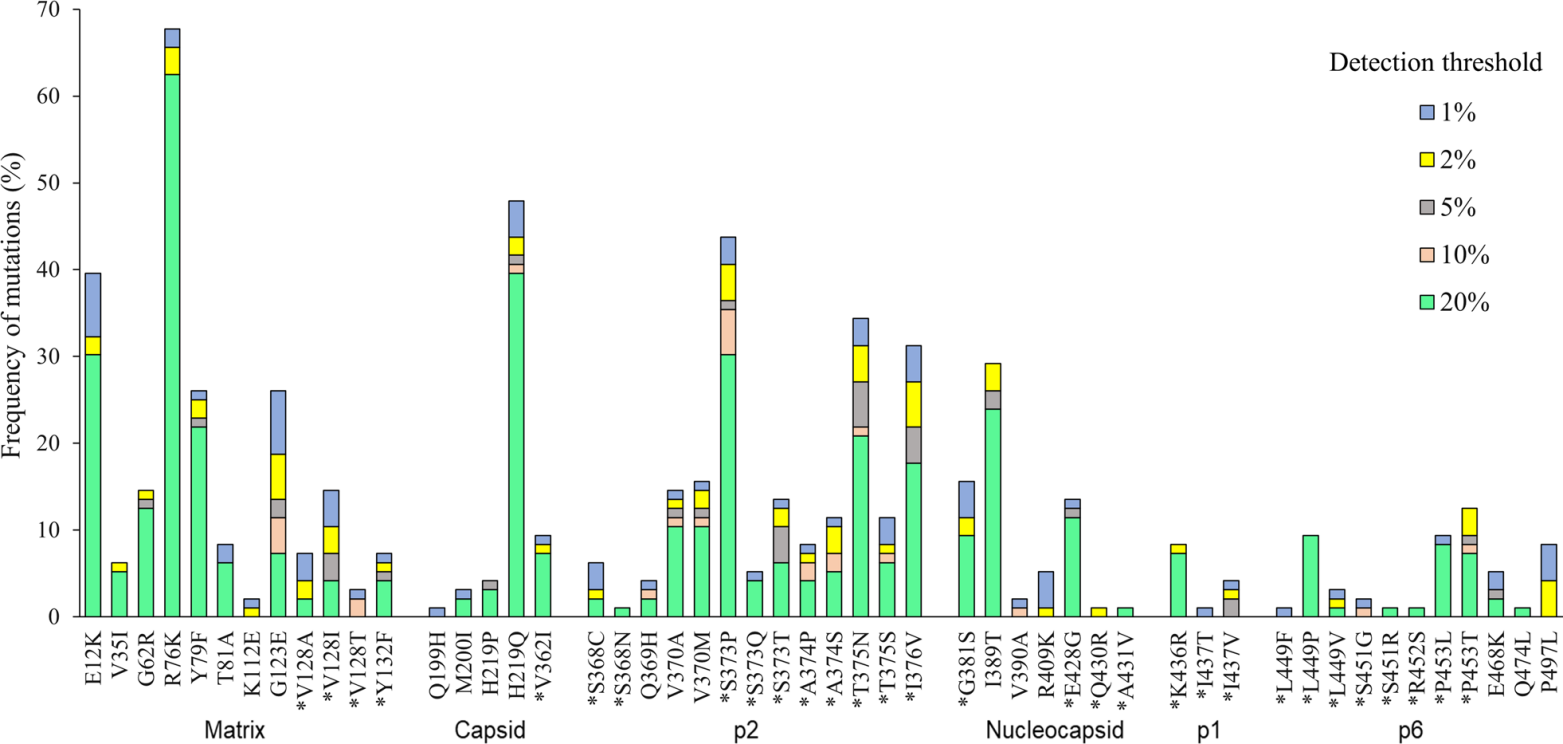
HIV Gag drug resistance-associated mutation frequency at different detection sensitivity thresholds (n = 96). Gag cleavage site mutations are indicated with an asterisk

Mutations associated with resistance to PI were found in Gag CS and NCS. We identified a total of 1184 mutations in Gag, of which 51 have been associated with resistance to PI, most were detected at the 20% threshold (Fig. 3). The mutation with the highest frequency at 1% detection threshold was R76K with 65.1% (54/83) in the MA protein, H219Q 44.6% (37/83) in CA protein, I389T 24.1% (20/83) in NC protein and P453T 8.4% (7/83) in p6 protein.

The median time to ART initiation was 38.5 days [IQR 20.8–60.5]. Data on the first-line ART regimen was available for 98 PLWH: 62 (63.3%) initiated with 2 NRTI + 1 NNRTI, 34 (34.7%) with 2 NRTI + 1 PI and 1 (1.0%) with 2 NRTI + 1 INSTI (Table 1). A total of 12 (12.2%) PLWH switched to second line ART, mainly due to side effects (Additional file 1). Follow-up HIV clinical variables (pVL and CD4 cell count) were available for 79 PLWH 6 months after ART initiation (Additional file 1). Sixty-three (80.8%) PLWH achieved complete viral suppression (defined as pVL < 50 copies/mL) while 72 (92.3%) achieved viral suppression (defined by pVL below 200 copies/mL after 6 months on ART). The median CD4 cell count was 437 cells/mm^3^ [IQR 230–614], with 31 (39.7%) PWLH achieving CD4 cell counts above 500 cells/mm^3^. Seven PLWH failed to suppress plasma viremia below 200 copies/mL (Additional file 1) and might be at risk of virologic failure, which would need to be confirmed. No major PI mutations were identified in these individuals at baseline. Treatment outcomes according to the presence of baseline Gag and protease drug resistance-associated mutations in PLWH are summarized in Table 3.

**Table 3:** Presence of drug resistance associated mutations after 6 months on ART

ART follow up n = 79	Parameters	*Without Baseline protease DRMs n (%)	*With Baseline protease DRMs n (%)	With < 5 Baseline Gag drug resistance-associated mutations n (%)	With ≥ 5 Baseline Gag drug resistance-associated mutations n (%)
2NRTI + 1PI 28 (35.4)	Viral load				
	< 50	4 (14.3)	17 (60.7)	4 (14.3)	17 (60.7)
	50—200	–	3 (10.7)	1 (3.6)	2 (7.1)
	> 200	1 (3.6)	2 (7.1)	–	3 (7.9)
2NRTI + 1NNRTI 50 (63.3)	Viral load				
	< 50	7 (14)	32 (64)	15 (30)	24 (48)
	50—200	–	5 (10)	–	5 (10)
	> 200	–	4 (8)	1 (2)	3 (6)

^*^Minor and major protease resistance mutations

*ART* antiretroviral therapy, *2NRTI + PI* two nucleoside reverse transcriptase inhibitors plus one protease inhibitor, *2NRTI + 1NNRTI* two nucleoside reverse transcriptase inhibitors plus one non- two nucleoside reverse transcriptase inhibitor, *DRMs* drug resistance mutations, *n* number, *%* percentage

## Discussion

HIV genotyping is a critical tool in HIV care and ART management, as it provides clinicians with relevant information to guide the choice for the most suited ART regimen, this can lead to ART success, together with treatment adherence. Previous studies in Mexico have analyzed DRMs in PLWH, but mainly in the HIV *pol* gene. In the present study we analyzed the presence of DRMs by NGS in the HIV protease and HIV Gag in ART-naive PLWH from the Hospital de Infectología, Instituto Mexicano del Seguro Social (IMSS), Mexico City. The Hospital de Infectología is a public national reference center that provides care to nearly 1,000 PLWH a month. Therefore, our cohort reflects a key population of Mexican PLWH, even though our study is not nationally representative. Demographic and clinical characteristics from our cohort were similar to those previously reported in Mexican PLWH including late stages of HIV disease and CD4 cell counts below 200 cells/ml at HIV diagnosis [36]. The prevalence of MSM was higher in the study compared to national reports (74.0% vs. 53.0%; 2016 national statistics).

Most sequences clustered with HIV-1 subtype B, as expected [10, 11], with 2 PLWH identified with HIV-1 non-B subtypes. In Mexico, the presence of inter-subtype recombinants (IRs) such as BG, BF, CRF12-BF and the CRF06-cpx have been reported [10, 37]. This can be explained by immigration (both temporary and permanent). In this context, Mexico is the second country of destination for migrants from South America [38] and also, Mexicans traveling abroad has increased [39]. The application of NGS techniques for HIV genotyping has been increasing; however, it is advisable to validate with Sanger sequencing [40]. Unfortunately, one of the limitations of this study was not performing Sanger sequencing, so we cannot validate our results (20% detection threshold). NGS techniques improve the detection of DRMs [8]; however, it has been suggested that low abundance variants detected below 5% threshold have little clinical relevance in treatment outcome [41]. In this study, we decided to use different detection thresholds from 1 to 20% with the purpose detecting a wide range of variants. Comparing to two recent studies [42, 43], we found higher rates of variation in the protease and in each Gag protein. Previous analysis of protease DRMs also reported a 3% of PI resistance with a threshold of 20% and 9.5% of resistance with a lower threshold (2%), this value was higher compared to our own results (4.2% of PI resistance) [11]. This difference may be due to NGS and bioinformatics pipelines. The most common major resistance mutation was M46I with 3.6% (threshold 1%) and 2.1% (threshold 2%) and I54V with 2.1% (20% threshold), frequencies that were higher compared to 1.5% for M46I (2% threshold) and 0.4% (20% threshold) detected in a previous study [11]. A study of amino acid variations of 777 Mexican protease sequences [12] showed similar presence of major and minor resistance mutations to protease inhibitors except for V11L, K43T, M46L, G48R, N88D, that were not detected in our study, however we were able to identify other mutations like M46I, L10F, K20T, M36I and I85V. We detected intermediate level of resistance for ATV and LPV, compared to a study reporting low level of resistance to PI [11]. Several Gag mutations in CS and NCS have been associated with resistance to PI [35]. We identified 51 Gag mutations in CS and NCS, with R76K being the most predominant at 65.1%. Resistance to PI and viral fitness, are often associated with a signature of mutations in Gag with the presence or absence of mutations in the protease [20]. A previous study found the R76K mutation was present in 57% in seven cases of virologic failure to boosted PI therapy [44]. Therefore, the high frequency of R76K mutation found in our study cohort is subject of concern. Our study showed that the p6 protein had the highest percentage of variation compared to the other Gag proteins and the mutation with the highest frequency was P453L/T. Mutations in P453 residue were found to enhance resistance to PI in the presence of PR mutations I50V and I84V [45, 46]. Six months after ART initiation, seven of the 79 PLWH for whom we had follow-up data, failed their first ART regimen. Although no major PI mutations were observed, several minor mutations in the protease and Gag associated resistance mutations were identified on these individuals. In this study, we identified several mutations in Gag proteins that to our knowledge have not been previously reported, such as H89Q, K418Q, I479M with a 17%, 18% and 21% frequency respectively. It would be interesting to perform phenotypic assays to determine if these mutations have a role in PI drug resistance.

Our study has several limitations, first the limited number of PWLH included in this study, and the lack of follow-up clinical data, which precludes analyzing the relationship between the presence of PR and Gag mutations and response to ART. We could not validate our results as Sanger sequencing wasn't performed. Next, we will address limitations inherent to using NGS to detected HIV DRMs as elegantly discussed by Ávila-Ríos and colleagues [41] and others [47, 48]. HIV genotyping results need to be carefully interpreted in light of biases and variations inherent to NGS. First, one limitation of our work is the absence of data on duplicate samples to determine how precise and reproducible our NGS results are, in particular for low frequency variants. Sequencing samples in duplicate was not possible due to budget limitations. We took all possible steps available to us to address some of these biases and variations, by using a high-fidelity PCR enzyme and following strict standard operating procedures. Also, we obtained a high coverage (> 7000 reads). Even though these steps were taken, we cannot exclude the possibility that artefactual sequence diversity might have been introduced during the entire process. The use of NGS for HIV genotyping has inherent drawbacks, which need to be addressed prior to its implementation in the clinical setting [41, 49]. For example, samples with low HIV RNA copy number or low input template (cDNA). A recent study has examined the effect of viral load on the detection of low abundant variants and found that these can only be detected in samples with higher plasma viral loads [50]. Our cohort was mostly composed of subjects with high plasma viral. Samples from 3 subjects had low plasma viral loads. One sample did not amplify, which proves the argument made on the effect of viral load and input template. Our ability to detect low variants in these two other samples might have been influenced by the low cDNA input. Also, important issues remain unanswered, such as the clinical utility and cost-effectiveness of HIV genotyping, in particular in low- and medium-income countries, where recommended first line ART regimens might not be available, yet HIV genotyping is prohibitive. Also, in countries where national policies are changing, or have recently changed like Mexico. In 2019, after the publication of several reports on the alarming increase in pretreatment drug resistance (PDR) [11, 51], and acting in accordance to the WHO´s recommendation to reconsider using NNRTIs in countries where levels of PDR to NNRTIs are high, Mexico updated their national antiretroviral treatment policy, recommending second-generation integrase strand-transfer inhibitor (INSTI)-based regimens as preferred first-line options since 2019. Our study focused on detecting the presence of DRMs by NGS in the HIV protease and HIV Gag in ART-naive PLWH from the Hospital de Infectología, Instituto Mexicano del Seguro Social (IMSS), Mexico City, given that at that time, PLWH were receiving PI as their first-line regimen. Also, there was limited data available on HIV-1 protease and gag mutations by NGS, which warranted the work presented here. Our results should be interpreted with caution due to the limitations in quality assessment already addressed, and should not be used to guide the choice of therapies for PWLH. Nonetheless, we believe our results provide interesting data that contributes to the ongoing discussion on adopting NGS for HIV genotyping.

## Conclusions

In conclusion, the present study reports the detection by NGS of drug resistant mutations in the Gag polyprotein and protease gene associated with protease inhibitor resistance in treatment-naive Mexican PLWH from The Hospital de Infectología del IMSS, a public national reference center that provides care to nearly 1,000 PLWH a month. The frequency of DRM in the HIV protease was 7.3% at a detection threshold of 1% and 3.1% at a detection threshold of 20%. Most of the mutations associated with resistance in Gag were detected at a threshold of 20%. Seven PLWH failed their ART regimen, however no major PI DRMs were found at baseline in these individuals.

## Supplementary Information

**Additional file 1: Table S1.** Primers used to amplify HIV-1 Gag and Protease genes. **Table S2.** GenBank accession numbers of the reference strains used in phylogenetic analysis. **Figure S1.** Sequencing depth of Gag and Protease sequenced genes per sample (n=96). **Figure S2.** Distribution of HIV Gag and Protease identified mutations from 96 treatment-naive patients. **Table S3.** Patients switching to second-line ART regimen. **Table S4.** Patient follow up data after 6 months of ART initiation according to plasma viral load ranges. **Table S5. **Baseline profile of HIV Gag and Protease drug resistance associated mutations and treatment outcomes in patients failing first line-ART.

## Data Availability

Sequences were deposited at the Sequence Read Archive under BioProject PRJNA823730.

## Abbreviations

3TC: Lamivudine
a TRAM: Target Restricted Assembly Method
ABC: Abacavir
ART: Antiretroviral therapy
ARV: Antiretroviral
ATV/r: Atazanavir/r
ATV/r: Atazanavir/ritonavir
BWA: Burrows-Wheeler Aligner
cDNA: Complementary DNA synthesis
CENSIDA: National Centre for AIDS Prevention and Control
CMNR: Centro Médico Nacional La Raza
CS: Cleavage site
DRMs: Drug resistance mutations
DRV/R: Darunavir/r
DTG: Dolutegravir
Dx: Diagnosis
EDTA: Ethylenediaminetetraacetic acid
EFV: Efavirenz
FIS: Fondo de Investigación en Salud
FTC: Emtricitabine
HIV: Human Immunodeficiency Virus
HIVDB: HIV Drug Resistance Database
HIVMMER: Hidden Markov Model for HIV
IMSS: Instituto Mexicano del Seguro Social
INSTI: Integrase inhibitor
IQR: Interquartile range
LPV/r: Lopinavir/ritonavir
MA: Matrix
n: Number
NC: Nucleocapsid
NCS: Non-cleavage site
NFV: Nelfinavir
NGS: Next-generation sequencing
NNRTI: Non-nucleoside reverse-transcriptase inhibitor
NRTIs: Nucleoside reverse-transcriptase inhibitors
PCR: Polymerase chain reaction
PI: Protease inhibitor
PLWH: People living with Human Immunodeficiency Virus
PR: Protease
pVL: Plasma viral load
SAM: Sequence Alignment/Map
SDRM: Surveillance drug resistance mutation
TDF: Tenofovir
WHO: World Health Organization
