# Supplementary material for: Protease and gag diversity and drug resistance mutations among treatment-naive Mexican people living with HIV
Source: BMC Infect Dis. 2022 May 10;22:447. doi: 10.1186/s12879-022-07446-8 (PMC9088029; doi:10.1186/s12879-022-07446-8)
Supplement: Supplementary file 1 — Additional file 1: Table S1. Primers used to amplify HIV-1 Gag and Protease genes. Table S2. GenBank accession numbers of the reference strains used in phylogenetic analysis. Figure S1. Sequencing depth of Gag and Protease sequenced genes per sample (n=96). Figure S2. Distribution of HIV Gag and Protease identified mutations from 96 treatment-naive patients. Table S3. Patients switching to second-line ART regimen. Table S4. Patient follow up data after 6 months of ART initiation according to plasma viral load ranges. Table S5. Baseline profile of HIV Gag and Protease drug resistance associated mutations and treatment outcomes in patients failing first line-ART. [file 12879_2022_7446_MOESM1_ESM.docx]

**Protease and Gag Diversity and Drug Resistance Mutations Among Treatment-Naive Mexican People Living with HIV**

Samantha Climaco-Arvizu^1,2*^, Víctor Flores-Lopez^3*^, Carolina González-Torres^4^, Francisco Javier Gaytán-Cervantes^4^, María Concepción Hernández-García^5^, Paola Berenice Zárate-Segura^2^, Monserrat Chávez-Torres^6^, Emiliano Tesoro-Cruz^1^, Sandra María Pinto-Cardoso^6§^, Vilma Carolina Bekker-Méndez^1§^

**Additional files**

Additional file 1. **Table S1 Primers used to amplify HIV-1 Gag and Protease genes.**

| **Process** | **Primer** | **Primer sequence 5’ to 3’** | **HXB2 position*** | **Mix (concentration/ volume)** | **Cycling conditions** | |
| --- | --- | --- | --- | --- | --- | --- |
| RT-PCR | KVL064 | GTTGTGTGACTCTGGTA  ACTAGAGATCCCTCAGA | 570 - 603 | Reaction Mix (1x)^1^ Template RNA (10μL) RNA Protector (10 U)^2^  MgSO_4_ (0.8 mM)^2^  Sense primer (0.2 μM)  Anti-sense primer (0.2 μM) SuperScript™ III RT/Platinum HF™ (1μL, 2,0 U)^1^  Water (qsp 50μL) | RNA incubation  65°C 30’’  55°C 5’  RT  55°C 30’  PCR cycling  94°C 2’ | |
|  | KVL065 | TCCTAATTGAACYTCC  CARAARTCYTGAGTTC | 2797 - 2828 |  | 94°C 15’’  57° 30’’  68°C 2’ | 40X |
|  |  |  |  |  | 4°C forever | |
| Nested PCR | KVL066 | TCTCTAGCAGTGGCGC  CCGAACAG | 626 - 649 | Mix1  dNTP (200mM)^1^  Sense primer (0.4μM)  Anti-sense primer (0.4μM)  RT-PCR product (5μL)  Mix 2  Buffer (1x)^2^  MgCl_2_ (2 mM)^2^  Expand HF-Enzyme (2,65 U)^2^  Water (qsp 25μL) | PCR cycling  95°C 2’ | |
|  | KVL067 | GGCCATTGTTTAACYT  TTGGDCCATCC | 2597 - 2623 |  | 95°C 15’’  58°C 30’’  68°C 2’30’’ | 10x |
|  |  |  |  |  | 95°C 15’’  58° 30’’  68°C  2’30’’+5/cycle | 30x |
|  |  |  |  |  | 4°C forever | |

Primers and cycling conditions are referenced from Van Laethem et al., 2006, Vrancken et al., 2016.

*Nucleotide position of the primer according to the HXB2 sequence (K03455) numbering. Forward primers: KVL064 and KVL066, reverse primers: KVL065 and KVL067.

RT-PCR= Reverse transcription polymerase chain reaction, PCR= Polymerase chain reaction, qsp= Quantity sufficient for the volume needed, dNTP= deoxyribonucleotidetriphosphate.

1 TermoFisher, Waltham, MA, USA,

2 Roche, Manheim, German

Additional file 1. **Table S2 GenBank accession numbers of the reference strains used in phylogenetic analysis.**

| **HIV Subtypes** | **Strains** | **Accession numbers** |
| --- | --- | --- |
| A1 | 3 | AB253421.1 AB253429.1 DQ676872.1 |
| A2 | 3 | AF286237.1 AF286238.1 GU201516.1 |
| B | 4 | AY173951.1 AY331295.1 AY423387.1 HIVHXB2CG |
| C | 4 | AF067155.1 AY772699.1 U46016.1 U52953.1 |
| D | 3 | AY371157.1 K03454.1 U88824.1 |
| F1 | 3 | AF005494.1 AF075703.1 AF077336.1 |
| F2 | 2 | AF377956.1 AY371158.1 |
| G | 2 | AF084936.1 AY612637.1 |
| H | 4 | AF005496.1 AF190127.1 AF190128.1 FJ711703.1 |
| J | 2 | AF082394.1 GU237072.1 |
| K | 5 | AJ249235.1 AJ249236.1 AJ249237.1 AJ249238.1 AJ249239.1 |

Abbreviations

HIV= Human Immunodeficiency Virus.

Additional file 1. **Figure S1 Sequencing depth of Gag and Protease sequenced genes per sample (n=96).**


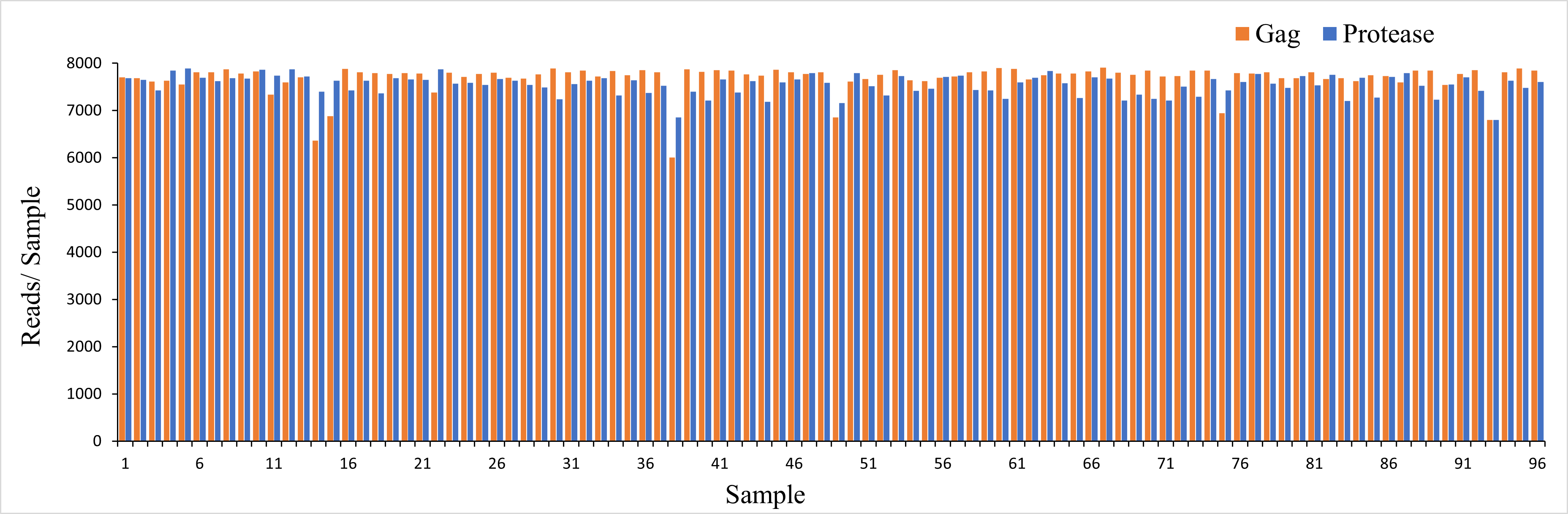


Legend: We sequenced the 1503 nucleotides corresponding to the full coding region of gag and 369 nucleotides corresponding to the 5’ end of the coding region of protease. Depth of coverage was calculated as the average number of reads, mapped to the corresponding region in the genome of HXB2 genome (gag: 790-2292; protease: 2253-2550). Bwa (28) was used to map such reads, and SAM tools (32) were used to calculate depth.

Additional file 4. **Figure 2 Distribution of HIV Gag and Protease identified mutations from 96 treatment-naive patients.**


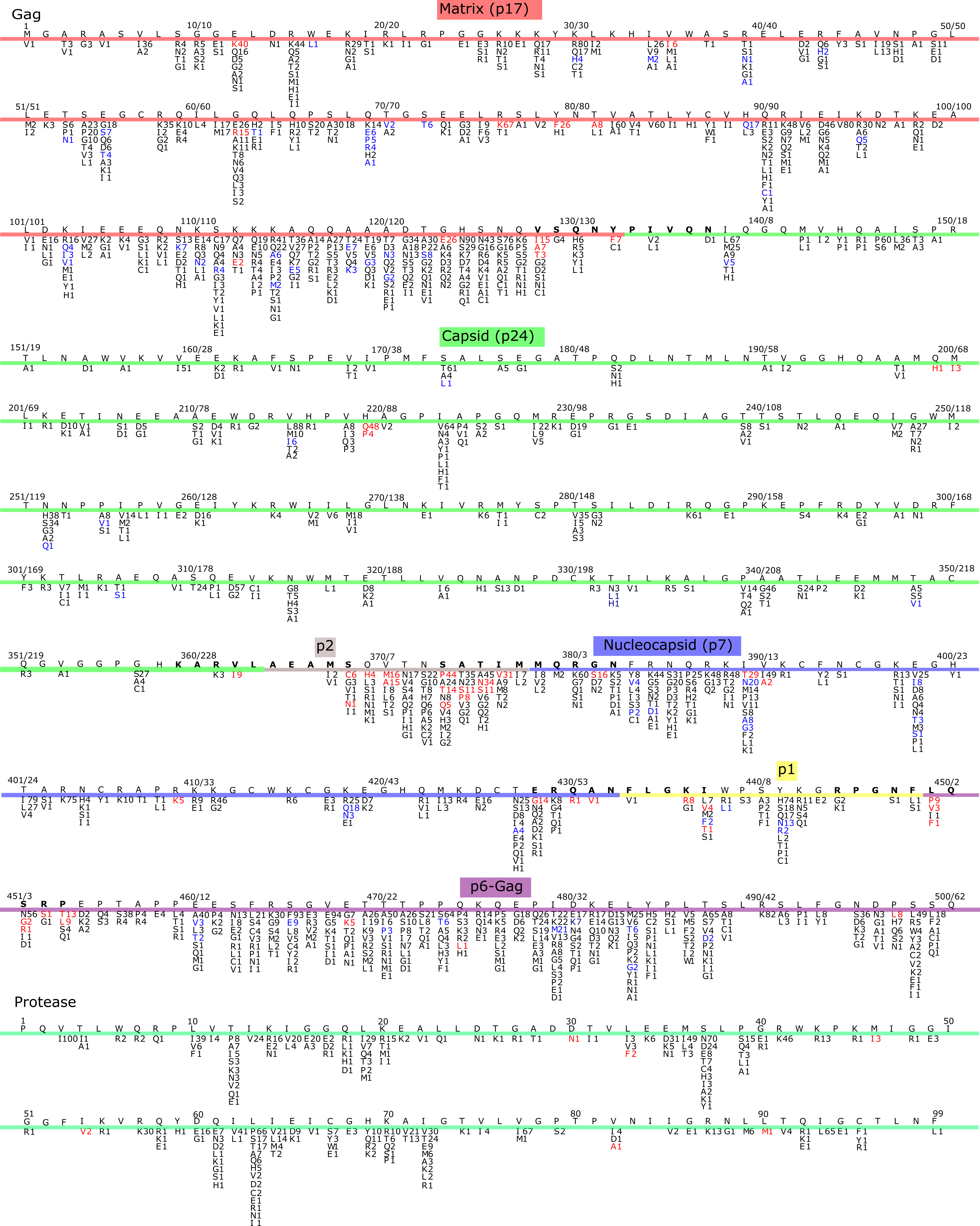


Legend: The reference sequence is found above the color bars, the 5 Gag cleavage sites are shown in bold, the identified mutations and their frequency (%) are followed by the reference residue. Mutations marked in red are those associated with resistance to PI, mutations marked in blue are those that so far to our knowledge have not been reported. Each protein is marked with a different color: pink for matrix (1-132), green for capsid (133-363), grey for p2 (363-377), blue for nucleocapside (378-432), yellow for p1 (433-448), purple for p6 (449-500) and light-blue for Protease (1-99).

Additional file 1. **Table S3 Patients switching to second-line ART regimen.**

| Patient | Time since ART initiation (days) | First-line ART regimen | Time to ART switch (days) | Second-line ART regimen | Reasons for switching ART |
| --- | --- | --- | --- | --- | --- |
| 1 | 32 | TDF-FTC-ATV/r | 169 | TDF-FTC-LPV/r | Virologic failure |
| 2 | 60 | TDF-FTC-EFV | 269 | TDF-FTC-ABC | Virologic failure |
| 20 | 83 | TDF-FTC-EFV | 17 | ABC-3TC-EFV | TDF hypersensitivity |
| 32 | 32 | TDF-FTC-ATV/r | 31 | TDF-FTC-EFV | Side effects: dizziness, painful urination |
| 38 | 92 | TDF-FTC-ATV/r | 29 | ABC-3TC-ATV/r | Generalized rash |
| 46 | 11 | TDF-FTC-LPV/r | 108 | TDF-FTC-EFV | Suspension due to appendicitis |
| 48 | 156 | TDF-FTC-EFV | 405 | TDF-FTC-EVG | EFV side effects |
| 53 | 37 | TDF-FTC-EFV | 207 | TDF-FTC-LPV/r | EFV side effects |
| 60 | 246 | TDF-FTC-EFV | 78 | TDF-FTC-RAL | Liver damage |
| 62 | 4 | TDF-FTC-EFV | 293 | ABC-3TC-EFV | TDF toxicity |
| 65 | 17 | TDF-FTC-EFV | 50 | ABC-3TC-EFV | Kidney failure |
| 93 | 7 | TDF-FTC-ATV/r | 32 | TDF-FTC-RAL | To avoid delirium |

Abbreviations

ART= antiretroviral therapy, TDF= Tenofovir, FTC= Emtricitabine, ATV/r= Atazanavir/ritonavir, EFV= Efavirenz, LPV/r=Lopinavir/ritonavir, ABC= Abacavir, 3TC= Lamivudine, RAL= Raltegravir, EVG= Elvitegravir.

Additional file 1. **Table S4 Patient follow up data after 6 months of ART initiation according to plasma viral load ranges.**

| Plasma viral load ranges | n (%) | Plasma viral load (copies/mL)  median [IQR] | CD4 count (cell/mm^3^)  median [IQR] |
| --- | --- | --- | --- |
|  | 79 (100%) | 40 [40-40] | 437 [230.0-614.0] |
| pVL 50 - 200 | 9 (11.4%) | 98 [62.5-105.5] | 278 [209.5-324.5] |
| pVL >200 | 7 (8.9%) | 655 [222-3250] | 462 [150.0-623.0] |

Abbreviations

n= number, %= percentage, IQR= interquartile range, pVL= plasma viral load.

Additional file 1. **Table S5.** **Baseline profile of HIV Gag and Protease drug resistance associated mutations and treatment outcomes in patients failing first line-ART.**

| **Patient** | **Treatment** | **Viral load** | | **CD4 count** | | **Total of Gag** | **Gag drug resistance** | **Protease minor** |
| --- | --- | --- | --- | --- | --- | --- | --- | --- |
|  |  | **Basal** | **After ART** | **Basal** | **After ART** | **mutations** | **associated mutations** | **mutations** |
| 1 | TDF-FTC-ATV/r | 280467 | 655 | 17 | 214 | 82 | V35I, G62R, R76K, H219Q, V370A, T375N | M36I |
| 2 | TDF-FTC-EFV | 270415 | 71 | 106 | 321 | 63 | E12K, G62R, R76K, E428G | L10I |
| 16 | ABC-3TC-ATV/r | 70995 | 204 | 374 | 705 | 62 | E12K, R76K, Y79F, V128I, H219Q | - |
| 23 | ABC-3TC-ATV/r | 6308 | 99 | 541 | 840 | 54 | E12K, S373P, A374P | M36I, A71V |
| 37 | TDF-FTC-ATV/r | 215192 | 99 | 71 | 307 | 98 | E12K, Y79F, G123E, V362I, I389T | L10I, M36I |
| 40 | ABC-3TC-ATV/r | 81392 | 112 | 152 | 275 | 80 | R76K, G123E, V370A, I376V, I389T | L10, V11I |
| 48 | TDF-FTC-EFV | 152058 | 288 | 329 | 509 | 91 | E12K, Y79F, H219Q, V370M, S373P, L449F | M36I, A71T |
| 53 | TDF-FTC-EFV | 3087 | 163322 | 127 | 132 | 74 | R76K, S373P | K20R, M36I |
| 58 | TDF-FTC-ATV/r | 281769 | 3250 | 11 | 623 | 92 | R76K, Y79F, K112E, H219Q, S373P, A374P, I376V, R409K, P453L, | L10I |
| 63 | TDF-FTC-EFV | 927678 | 98 | 31 | 278 | 53 | R76K, Y9F, V128I, H219Q, V362I, I389T | L10I |
| 64 | TDF-FTC-EFV | 723642 | 157 | NA | 273 | 72 | Y79F, H219Q, A374S, S451G, P453T | M36I |
| 68 | TDF-FTC-EFV | 1262814 | 54 | 24 | 328 | NA | NA | NA |
| 76 | TDF-FTC-EFV | 675322 | 222 | 90 | 150 | 130 | E12K, V35I, R76K, Y79F, T81A, V128A/T, Y132F, H219Q, V362I, S368C, G381S | M36I |
| 80 | TDF-FTC-EFV | 85583 | 1011 | 493 | 462 | 121 | G62R, R76K, Y132F, H219Q, V370M, S373P, A374P, T375N, I376V, I389T, R409K, P453T | K20R, M36I, A71T |
| 83 | TDF-FTC-EFV | 145842 | 50 | 68 | 146 | 95 | R76K, V128I, H219Q, S373P, A374P, T375N, I389T | M36I |
| 84 | TDF-FTC-EFV | 181371 | 80 | 32 | 112 | 84 | R76K, Y132F, H219Q, V362I, S373P, T375N/S, G381S, E468K, P497L | M36I |

Abbreviations

ART= antiretroviral therapy, TDF= Tenofovir, FTC= Emtricitabine, ATV/r= Atazanavir/ritonavir, EFV= Efavirenz, ABC= Abacavir, 3TC= Lamivudine, NA= Not available.
